# Supplementary figures and images for: Enhancement of specific T-lymphocyte responses by monocyte-derived dendritic cells pulsed with E2 protein of human papillomavirus 16 and human p16INK4A
Source: PeerJ. 2020 May 20;8:e9213. doi: 10.7717/peerj.9213 (PMC7245333; doi:10.7717/peerj.9213)

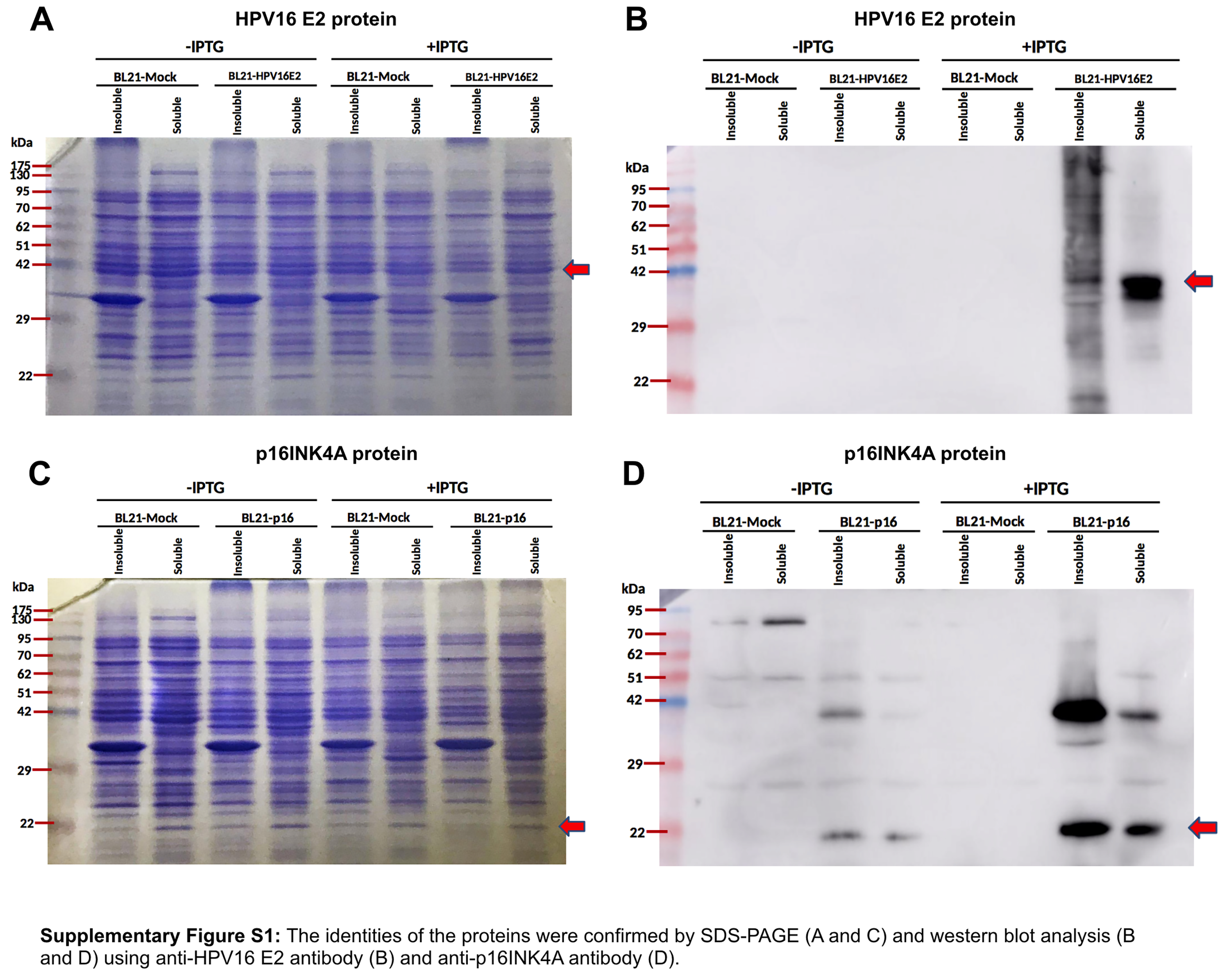

Supplement: Supplemental Information 1 [file peerj-08-9213-s001.png]
